# Non-Invasive Measurement of Cortical Plasticity in Brain Tumour Surgery: A Monocentric Experience of nTMS Mapping and Definition of Cognitive Reshaping Based on Tumour Histological Grade

**DOI:** 10.3390/cancers18091405

**Published:** 2026-04-28

**Authors:** Camilla Bonaudo, Matteo Elias Schapira, Edoardo Pieropan, Charly Caredda, Eric Van Reeth, Francesca Fedi, Elisa Castaldi, Fabrizio Baldanzi, Simone Troiano, Antonio Maiorelli, Agnese Pedone, Eleonora Visocchi, Bruno Montcel, Riccardo Carrai, Antonello Grippo, Luca Campagnaro, Serena Tola, Alessandro Della Puppa

**Affiliations:** 1 Department of Neuroscience, Psychology, Pharmacology and Child Health, University of Florence, Viale Pieraccini 6, 50139 Florence, Italy; camilla.bonaudo@unifi.it (C.B.);; 2 Neurosurgery, Department of Neuroscience, Psychology, Pharmacology and Child Health, University Hospital of Careggi, University of Florence, Largo Brambilla n 3, 50134 Florence, Italy; 3 Antares S.p.A., Via Prati 11, Dueville, 36031 Vicenza, Italy; 4 CREATIS UMR 5220, U1294, INSA-Lyon, Université Claude Bernard Lyon 1, CNRS, Inserm, 69100 Lyon, France; 5 Département Sciences du Numérique, CPE Lyon, Rue Victor Grignard, 69100 Villeurbanne, France; 6 Neurophysiopathology Unit, University Hospital of Careggi, University of Florence, 50139 Florence, Italyantonello.grippo@unifi.it (A.G.)

**Keywords:** brain plasticity index, navigated transcranial magnetic stimulation (nTMS), glioma, functional reorganisation, cortical plasticity, awake surgery, DTI tractography, cognitive mapping

## Abstract

**Background and Objectives:** Cortical plasticity assessment using navigated transcranial magnetic stimulation (nTMS) represents a promising non-invasive strategy for predicting reorganisation of cortical circuits in neuro-oncological patients. This study examined how glioma grade influences cognitive network reorganisation by multiparametric analysis. **Materials and Methods:** We conducted a prospective monocentric study at the Neurosurgical Department in Florence and a comparative analysis of motor (M), language (Ln), calculation (C), and visuo-spatial functions (VS) between patients with low-grade gliomas (LGGs) and high-grade gliomas (HGGs) undergoing pre- and postoperative nTMS mapping (at 5 ± 2, 30 ± 10, and 90 ± 10 days of follow-up). **Results:** Between January2024 and September 2025, we enrolled 69 patients, and the total number of nTMS mapping procedures was 70: one relapse, (M:F = 345:365), level of scholarship 8–15 years, 21 LGGs, 30 HGGs, 19 non-glial lesions (excluded), left lesions *n* = 37, right lesions *n* = 31, bilateral *n* = 2, bi-hemispheric nTMS = 80%. Considering LGGs and HGGs, the major motor function displacement was obtained in the right hemisphere (Rh; predominantly for HGGs 64 mm vs. LGGs 39 mm), with more restrained displacement in the left hemisphere (Lh; LGGs 20 mm vs. HGGs 21 mm). For Ln, displacement was higher for HGGs (57 mm vs. LGGs 31 mm). However, surprisingly for HGGs in the Lh, the displacement was more significant (60 mm), whereas for LGGs it was major in the Rh (~80 mm). For C, displacement for HGGs was 72 mm Lh vs. 48.11 mm Rh, and for LGGs 50 mm Lh vs. 41 mm Rh. Insufficient data were obtained for the network. Qualitative analyses further characterised this reorganisation: motor f. demonstrated reshaping around the primary motor cortex; linguistic f. displaced from temporo-parietal areas to the inferior frontal gyrus; calculation and VS functions reorganised within frontoparietal circuits. The correlation between cognitive results and BPI revealed that higher BPI values were associated with prolonged recovery periods. Nevertheless, functional recovery was achieved in up to 90% of patients across all assessed functions. **Conclusions:** We propose non-invasively measuring cortical plasticity across different cognitive domains with a quantitative–qualitative framework for assessing functional reorganisation with a multimodal assessment in glioma patients.

## Simple Summary

This prospective monocentric study investigated cortical plasticity in glioma patients using navigated transcranial magnetic stimulation (nTMS). A total of 69 patients with low-grade gliomas (LGGs) and high-grade gliomas (HGGs) underwent pre- and postoperative mapping of motor, language, and calculation functions at multiple follow-up intervals. The results showed greater functional displacement in HGGs compared to LGGs, particularly in motor and language networks, with hemispheric differences in reorganisation patterns. Qualitative analysis revealed functional reshaping around key cortical regions, including the motor cortex and frontoparietal networks. Higher brain plasticity index (BPI) values were associated with longer recovery times, although up to 90% of patients achieved high functional recovery. Overall, this study provides a multimodal, non-invasive framework to characterise cortical reorganisation across cognitive domains in neuro-oncological patients.

## 1. Introduction

Brain plasticity has become a central concept in contemporary neurosurgical oncology and plays a critical role in therapeutic decision-making for glioma patients [1,2]. Modern surgical strategies are guided by a function-based, patient-centred paradigm grounded in the principle of onco-functional balance [3,4,5,6,7], which aims to maximise tumour resection while preserving neurological function and quality of life (QoL) [7,8]. This approach relies on functional mapping to account for the substantial interindividual variability in the structural and functional organisation of brain networks observed in glioma patients [2,9,10]. Accordingly, the modern ‘functional approach’ may lead neurosurgeons to intentionally leave residual tumour tissue when critical cortico-subcortical structures essential for neurological function are involved, or conversely to pursue supramarginal resections when functional networks are spatially remote from the lesion [8]. The possibility of achieving maximal safe resection is closely linked to neuroplasticity, defined as the brain’s capacity for functional reorganisation in response to injury or lesions [2,11]. Characterising the individual profile of neural reconfiguration is consequently crucial for tailoring therapeutic strategies that promote adaptive plastic mechanisms [1,8]. Within this framework, the concept of meta-plasticity, or the ‘plasticity of plasticity’, describes higher-order regulatory mechanisms that modulate the brain’s capacity for adaptive change according to contextual demands. This perspective is consistent with the emerging view of gliomas as chronic diseases of the connectome [12] in which tumour growth progressively interacts with distributed functional networks rather than isolated cortical regions in a dynamic context [13]. Neuroplastic potential varies across different neural substrates. Cortical regions exhibit substantial plastic capacity [14], allowing functional reorganisation through recruitment of adjacent or distant cortical areas following injury [15]. Conversely, white-matter tracts demonstrate lower interindividual variability and limit capacity for reorganisation, resulting in more restricted subcortical plasticity [16]. Consequently, preservation of axonal connectivity is a critical prerequisite for enabling compensatory neuroplastic processes and maintaining functional network integrity [15].

Among the available mapping techniques, non-invasive transcranial magnetic stimulation (nTMS) has emerged as a reliable method for preoperative mapping of motor and higher-order cognitive functions [17,18,19,20,21]. nTMS offers several practical advantages, including non-invasiveness, bilateral hemispheric mapping capability, applicability in routine clinical settings, and a favourable safety and tolerability profile [19,22]. Integrated with patient-specific 3D MRI data, nTMS enables precise preoperative functional mapping and can guide DTI-based tractography to reconstruct motor- and language-related subcortical pathways [23,24,25]. As a planning tool, nTMS supports safer and more personalised surgical strategies and shows strong concordance with direct cortical stimulation (DCS) performed during awake surgery [20]. Importantly, nTMS also represents a valuable alternative for functional assessment when awake procedures are not feasible, thereby expanding the potential for safe tumour resection [25]. Previous work from our group demonstrated that pre- and postoperative nTMS data can be used to quantify cortical language organisation, enabling the development of a brain plasticity index (BPI) that may be extended to multiple cognitive domains [26]. Functional mapping should be complemented by a comprehensive neurocognitive assessment, typically performed by neuropsychologists and speech therapists at pre-, intra-, and postoperative stages. Global cognitive status can be evaluated using the Mini-Mental State Examination (MMSE) [27], while language functions are assessed through standardised instruments, such as the Aachener Aphasia Test (AAT) [28] for the assessment of language deficits in acquired aphasia, ELLM (Italian acronym for “language assessment at the patient’s bedside”) [29], and Neuropsychological Examination for Aphasia (ENPA) [30].

Visuo-spatial functions may be evaluated using the Oxford Cognitive Screen (OCS), ‘Broken Hearts’ cancellation task [31], Bell cancellation test [32], and Clock drawing test [32,33]. Finally, executive functions can be screened using the Frontal Assessment Battery (FAB) [34].

Within this framework, the present study aimed to advance surgical planning in glioma patients by integrating tumour classification, advanced functional mapping, and quantitative measures of brain plasticity. Specifically, we investigated how glioma grade influences cognitive network reorganisation in patients undergoing nTMS-based mapping. We hypothesised that nTMS combined with diffusion tensor imaging (DTI) tractography and comprehensive neurocognitive evaluation provides a robust platform for the pre- and postoperative assessment of neuro-oncological patients. Additionally, we explored whether strategy (awake versus asleep procedures) influences cognitive outcomes. This integrated approach may facilitate high-quality functional neurosurgery while enabling the development of individualised postoperative rehabilitation strategies informed by patient-specific plasticity metrics.

## 2. Materials and Methods

A monocentric prospective clinical observational study was designed and approved by the CEAVC Ethics Committee (Comitato Etico Area Vasta Centro (CEAVC) protocol 17003), a section of the Regional Ethics Committee of Tuscany, in accordance with the principles of the Declaration of Helsinki. We selected oncological patients followed at the Department of Neurosurgery of the University of Florence harbouring primary brain lesions (intra- or extra-axial), vascular lesions (i.e., cavernomas/lymphomas), or focal cortical dysplasia (FCD).

All data were systematically organised into an Excel spreadsheet (Table of patients’ characteristics attached in the Supplementary Material).

Patients underwent a preoperative logopaedic/neurocognitive evaluation with nTMS motor and cognitive mapping around 48–72 h before surgery. Considering the location and nature of the lesion, an awake or asleep procedure was chosen based on a multidisciplinary team evaluation. A postoperative re-evaluation was performed immediately after surgery, with follow-up (FU) assessments conducted at (I) 5 ± 2 days, (II) 30 ± 10 days, and (III) 90 ± 10 days postoperatively with repetition of nTMS (at the first and/or second FU). Data from cognitive assessments (language (Ln), calculation (C), and visuo-spatial (VS) functions) were collected, along with pre- and postoperative imaging data. Evaluations of logopaedic/cognitive tasks, performed by F.F., were also analysed for each specific item.

Pivot tables were generated from the Excel dataset, comparing patients with low-grade (LGGs) and high-grade gliomas (HGGs) as well as those with non-glial lesions (the latter excluded from the main analysis), and differentiating between left hemisphere (Lh) and right hemisphere (Rh).

The aim of surgery was to achieve a gross total resection (GTR), i.e., tumour resection > 90% of lesion volume [35], and subtotal resection was performed only when functional boundaries were encountered to avoid impairment.

### 2.1. Clinical Characteristics and Radiological Classification

For each patient, clinical-anamnestic data were collected. Based on the anaesthetic modality, surgeries were classified as ‘awake’ or ‘asleep’ (recorded under the variable ‘stato int’). See Table 1.

Tumour location is categorised under ‘Lobe’ (frontal—F, temporal—T, parietal—P, occipital—O, insular—In), and lesion side was indicated in the ‘Site’ column (left—L; right—R). Tumours were classified according to the WHO 2021 classification, with LGGs (G2) coded 2 and HGGs (G3 and 4) 4. IDH mutation status (‘1’ = mutant; ‘0’ = wild type) was also reported, and the ‘Histology WHO 2021’ [36] column indicates the final histopathological diagnosis.

### 2.2. Timing of Assessments

Each patient underwent functional assessments at three time points: preoperative, early postoperative, and late FU. The timing of each phase was recorded as ‘Data Int’ (date of surgery) and ‘Data TMS’ (date of preoperative nTMS), as well as ‘Data Post +’ (5 ± 2 days after surgery) and ‘Data Post ++’ (at 30 ± 10 days and/or 90 ± 10 days after surgery). Cognitive test columns were labelled with the test name, followed by ‘+’ or ‘++’ to indicate early postoperative or late FU assessments, respectively.

### 2.3. Functional Assessment

The protocol involved the evaluation of several higher-order cortical functions, with a focus on language, arithmetic and visuo-spatial abilities, and global cognition. The objective was to examine the main cognitive-linguistic domains potentially affected by the tumour or surgery. All tests were performed by our speech therapist (FF).

Global executive functioning in patients with frontal cortex lesions was assessed using the FAB [34]. Of the 73 patients selected, only 30 underwent testing. For each temporal phase, two scoring indices were reported: PC (punteggio corretto—corrected raw score) and PE (punteggio equivalente—equivalent score).

General cognitive status was evaluated at each stage using the Mini-Mental State Examination (MMSE) [27].

Language (Ln): Spontaneous speech (LS) was assessed using the Aachener Aphasie Test (AAT) [28], which evaluates six parameters on a 0–5 scale: general communicative abilities (LS: Ab Com), articulation and prosody (LS: Art Pros), lexical–semantic processing (LS: Les Sem), automatic language (LS: L Auto), phonology (LS: Fono), and morphosyntax (LS: Morf Sint). In some cases, the ELLM test (Esame del Linguaggio al Letto del Malato [29]) was used.

Calculation (C): We applied basic operations such as addition (SOM), subtraction (DIF), and multiplication (MOLT) using the Neuropsychological Test for Aphasia (ENPA) [30], with ‘1’ = preserved function and ‘0’ = impairment.

Visuo-spatial function (VSf): This was evaluated through three standardised tests—the Broken Hearts cancellation subtest of the Oxford Cognitive Screen (OCS) [37,38,39], the Bell test (BELL) [32], and the clock drawing test [33]—based on years of education and level of scholarship. Performance was classified ‘1’ for preserved function and ‘0’ for impairment.

### 2.4. Imaging and MRI Sequences

Preoperative magnetic resonance imaging (MRI) was performed at our Neuroradiology Department using a 3T scanner (Ingenia 3T, Philips Medical Systems, Best, The Netherlands), including diffusion tensor imaging (DTI) sequences. Multiple regions of interest (ROIs), defined according to preoperative nTMS mapping, were employed to reconstruct subcortical pathways. Motor function was assessed clinically using the Medical Research Council Scale [39] to evaluate muscle strength in both upper and lower limbs.

### 2.5. nTMS Stimulation and Data Acquisition

Preoperative cognitive mapping was performed with an rnTMS system (Galileo NetBrain Neuronavigator 9000, EB Neuro Corp., Florence, Italy). Navigated transcranial magnetic stimulation (nTMS) was employed to functionally map eloquent cortical areas both preoperatively and postoperatively. For each patient, data were collected on the tested functions (‘Functions’), indicating the mapped domains (motor, language, calculation, neglect), and on the stimulated hemisphere (‘Emis’), specifying whether it was left, right, or bilateral. For nTMS data acquisition, we used the same protocol as established in our previous studies [21,26,37,40]. Language mapping was carried out using the DO80 object-naming task combined with repetitive nTMS pulse trains (5 pulses/5 Hz), an inter-picture interval (IPI) of 1000 ms, and a picture presentation time (PPT) of 4000 ms. For arithmetic tests [21] and VS functions, the VISA test was used [37].

### 2.6. Pre- and Postoperative nTMS Mapping Data Image Processing Pipeline

The methodology for creating an imaging pipeline has been described in our research group’s previous work [21,26]. The method is implemented in Python (version 5.4) using open-source frameworks and incorporates the spatial coordinates of both positive and negative nTMS points. The BPI is computed as a displacement metric to quantify functional reorganisation. The resulting deformation map reflects the magnitude of displacement (in mm) required to align preoperative volumes with postoperative volumes defined by the distribution of nTMS stimulation sites.

### 2.7. Brain Plasticity Maps

#### 2.7.1. Quantitative Displacement

BP maps were then registered to a neuroanatomy atlas (NMI) space using a combination of affine and non-rigid (B-spline) registration procedures. The tumour segmentation was performed with the BRATS toolkit. Then, a normalised BP map for all patients was defined by the weighted sum of all BP maps [26].

#### 2.7.2. Qualitative Displacement

The Harvard–Oxford atlas was overlaid onto the cortical surface to enable precise identification of anatomical regions. Atlas-based parcellations were visually mapped onto the left and right hemispheres, allowing localisation of pre- and postoperative nTMS clusters within specific cortical areas. All statistical analyses and bioengineering procedures were conducted and supervised by Dr. E.P. and Dr. C.C. in accordance with the described pipeline. BPI results were then correlated with cognitive performance measures, including MMSE scores, as well as language, arithmetic, and visuo-spatial assessment.

#### 2.7.3. Statistics

We used box plots with interquartile ranges (IQRs) between 25% and 75%, meaning that the box contains the middle 50% of the data (either for LGGs and HGGs for specific functions we analysed). IQR (25–75%) describes the spread of the data and is based solely on observed values. Concerning evaluation of cognitive performances, we reported in Excel files the median values of cognitive scores in lines, whereas in columns we put the different time during follow-up for evaluation: the average of scores was calculated and reported in the columns to graphically design the trend of functional variation, either among HGGs and LGGs, or for awake/asleep procedures (qualitative and descriptive analyses).

### 2.8. Intraoperative Monitoring and Anaesthesiologic Parameters for Awake Surgery

We used the same protocol already proposed in previous research [21,26,37].

## 3. Results

Between January 2024 and September 2025, we selected 69 patients who had either pre- or postoperative mapping to study functional reshaping. They were operated on at the Neurosurgical Department of Careggi in Florence and underwent nTMS motor and cognitive preoperative mapping. The functional mapping was integrated into the neuronavigation system used during surgery, relying especially on the negative predictive value of nTMS, to spare active areas [17,19,21,24,37,41,42,43]. Mapping with nTMS was also used for tractography subcortical bundle visualisation. The final study cohort consisted of 69 patients, while the total number of nTMS mapping procedures was 70, since one patient underwent two surgeries due to relapse of a grade II astrocytoma.

General characteristics: The gender distribution was balanced (M:F = 34: 36), and the mean level of education ranged between 8 and 15 years: 21 LGGs (grade 2 s WHO 2021 [36]) and 30 HGGs (grade 3–4 s WHO 2021). Within the latter group, we enrolled 10 IDH-mutant astrocytomas, 16 IDH wild-type glioblastomas, two gliosarcomas, and two oligodendrogliomas. In addition, 19 patients had non-glial lesions and were excluded.

A total of 70 surgical procedures were performed: 47 under general anaesthesia (asleep surgery, 67.14%) and 23 under awake surgery (32.86%). Among these, 60 were first interventions (85.71%), while 10 were second interventions (14.29%). For one patient (P40/P41), nTMS data were collected for both the first and the second operation. For nTMS mapping, 55 procedures (78.57%) involved bilateral mapping, 11 (15.71%) left hemispheric mapping, and 3 (4.29%) right hemispheric mapping.

Of the 69 patients enrolled for 70 nTMS procedures, only those who completed all three phases of the cognitive assessment (preoperative, early postoperative, and late FU) conducted by the speech therapist were considered for the study. In total, 62 patients met these criteria. BPI was calculated according to our pipeline, resulting in a final number of 54 patients for whom pre- and postoperative maps were comparable. Based on the Excel sheet used to collect cognitive test scores, we generated pivot tables to compare the mean scores between LGGs and HGGs, as well as to analyse differences between patients who underwent awake vs. asleep surgery. Regarding VS functions, across the three study phases, not all patients completed all three tests for neglect (Bell, OCS, and Clock). Complete data were available for 25 out of the 69 patients.

### 3.1. Comparison Between LGGs and HGGs

**Figure 1:**
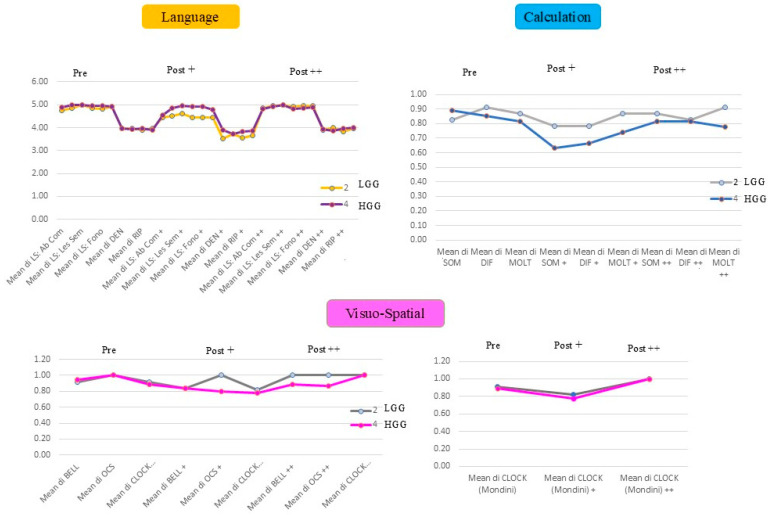
Mean scores derived from language, arithmetic, and VS assessment tests administered by the speech therapist. The graphical representation employs a color-coded system: low-grade gliomas (2) high-grade gliomas (4). (+ refers to the first postop evaluation, ++ refers to the long-term follow-up period).

A systematic comparison of cognitive performance between patients LGGs vs. HGGs across distinct cognitive domains is presented in Figure 1, with mean scores derived from Ln, C, and VS assessment tests.

For Ln, a similar trend was observed for LGGs and HGGs in terms of functional performance and recovery during the FU period. A comparable trajectory was observed in both LGGs and HGGs for C: a slight decline in C performance during the early postoperative FU, followed by partial improvement in the late postoperative phase. However, neither complete recovery nor marked enhancement was achieved. Mean values in HGGs consistently remained lower than those in LGGs, with the sole exception of the mean SOM score in the preoperative phase. Notably, within the LGG cohort, MOLT was the only subfunction to demonstrate improvement relative to preoperative baseline performance.

For VSf, the graph representation is based on the 25 patients who had all the scores of all three tests during the three phases. The graph demonstrates comparable mean values in the preoperative phase. Following surgery, a slight decline is observed in both LGGs and HGGs, except for OCS, which maintains full scores in LGG patients. However, whilst LGG patients demonstrate complete recovery at one month after surgery, HGG patients exhibit only partial recovery, with the notable exception of the clock drawing test, where performance improves more substantially. Finally, for the clock drawing, every patient was tested (except for P24): LGG and HGG patients’ performance were comparable over time (bottom-right graph, Figure 1).

### 3.2. Comparison Between LGGs and HGGs Operated on Asleep or Awake

We analysed the functional performance comparing awake vs. asleep procedures, differentiating between LGGs and HGGs, stratified across three cognitive domains. The graphical representation employs a colour-coded system: light blue indicates LGGs operated on under general anaesthesia (asleep surgery), orange represents LGGs operated on awake, dark blue denotes HGGs operated on under general anaesthesia, and red signifies HGGs operated on awake. The transient functional decline associated with awake surgery is likely since resection can be safely pushed closer to eloquent cortical areas and subcortical tracts, thereby inducing temporary functional disruption that does not preclude subsequent recovery, as previously demonstrated [44].

**Figure 2:**
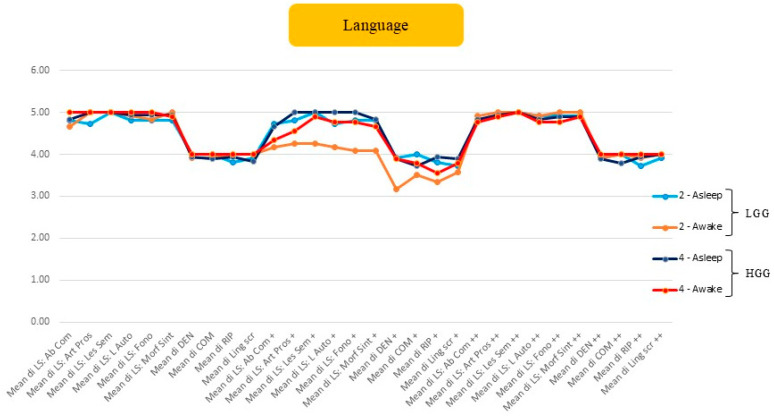
Changes in cognitive function scores across the language functions evaluated by the speech therapist for each phase of the study: spontaneous language (LS), denomination (DEN), comprehension (COM), repetition (RIP [M1] [CB2]), and written language (Ling Scr). Both types of gliomas (LGGs and HGGs) were evaluated according to the type of surgery performed: awake or asleep. (+ refers to the first postop evaluation, ++ refers to the long-term follow-up period).

***Language:*** Figure 2 illustrates the mean performance trend. Overall, we observed that in the preoperative phase, there was no significant difference in mean scores between LGGs and HGGs, with slightly lower values recorded for LGGs. In the post+ follow-up (FU) phase, however, a decline in cognitive scores was evident in patients operated on awake, particularly amongst LGGs. Subsequently, in the post++ phase, a marked improvement in cognitive test performance was observed, with comparable values between LGGs and HGGs, though superior values for LGGs.

**Figure 3:**
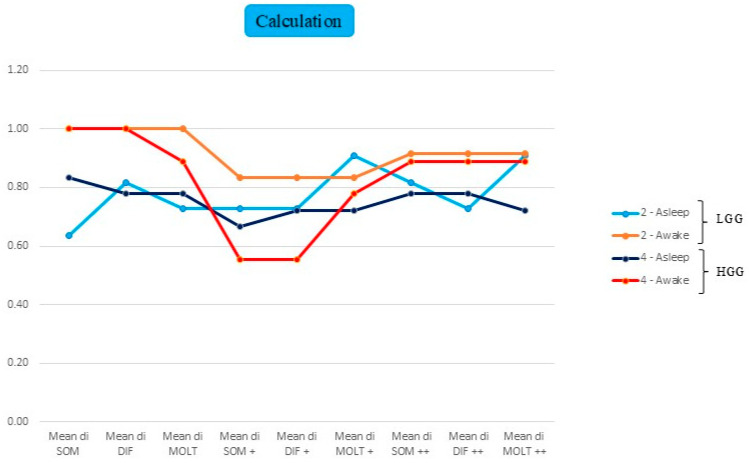
Comparison between low-grade (grade 2) and high-grade (grade 4) gliomas, illustrating different trends depending on the type of surgery (awake or asleep). The figure shows changes in cognitive function scores across three arithmetic tasks—addition (SOM), subtraction (DIF), and multiplication (MOLT)—over the three phases of the study. (+ refers to the first postop evaluation, ++ refers to the long-term follow-up period).

***Calculation:*** Figure 3 depicts the mean scores obtained in arithmetic assessments. When comparing patients according by the type of mathematical operation, those who underwent awake surgery exhibit similar trends: excellent baseline performance, a deterioration in the early postoperative FU at ~4 days, followed by marked improvement at the one-month FU, without complete recovery to preoperative function. Comparing LGGs and HGGs, whilst the trend remains remarkably similar, mean values for HGGs are almost invariably slightly lower, particularly for addition (SOM+) and subtraction (DIF+) in the early postoperative period, wherein a substantial decline in test scores is observed. Conversely, patients operated on asleep maintained consistently lower mean values and demonstrated fewer temporal variations. Among these, LGG values are more variable: the addition (SOM) and multiplication (MOLT) functions show modest improvement following surgery, which persists into the late-FU period without further significant gains. In contrast, the subtraction function (DIF) exhibits a slight deterioration postoperatively, evident in both the early (post+) and late (post++) FU periods. HGGs, however, display results that remain relatively stable over time both before and after surgery, ranging approximately between 70% and 85% of functional performance.

**Figure 4:**
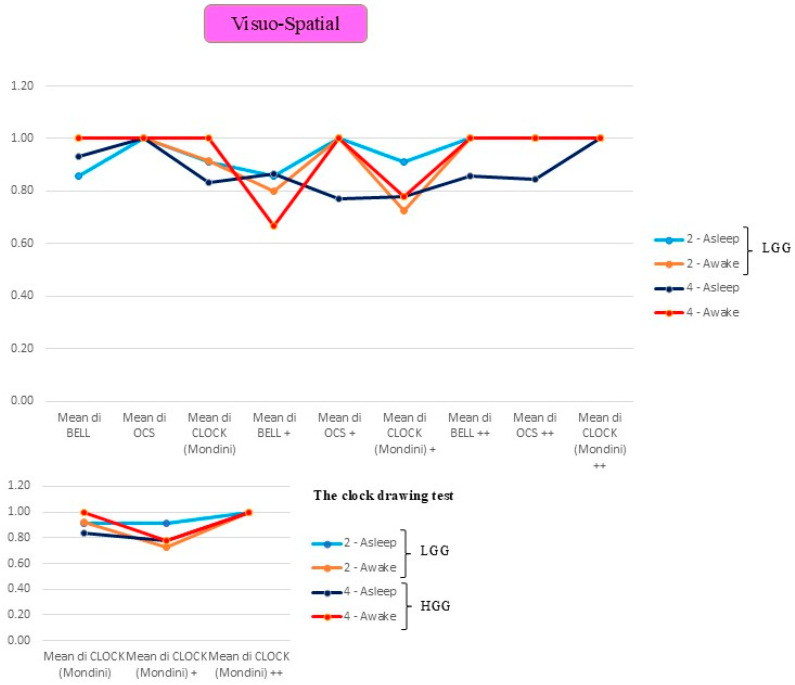
The tests for VS functions were the Bell test, Broken Hearts (OCS), and the clock drawing test (CLOCK Mondini), each tested across the three phases of the study, illustrating different trends according to the type of surgery (awake or asleep). The lower part of the figure shows the trend of the mean value of the clock drawing test across the three phases of the study, illustrating different trends according to the type of surgery (awake or asleep). (+ refers to the first postop evaluation, ++ refers to the long-term follow-up period).

***Visuo-spatial attention:*** Figure 4 represents the mean values for VSf/neglect function tests. The tests evaluated were the Bell test, Brocken Hearts (OCS), and the clock drawing test (CLOCK Mondini), each tested across the three phases of the study, illustrating different trends according to the type of surgery (awake or asleep). As observed in the analysis of other cognitive functions, patients who underwent awake surgery exhibit similar trajectories for both LGGs and HGGs: excellent preoperative test performance, a decline in the early postoperative period (except for the OCS test), and complete recovery of function in the late postoperative FU. Slight differences in mean values were noted in the early postoperative FU (post+), with lower values for HGGs in the Bell test, and minimal variation in the clock drawing test, wherein LGG patients demonstrated marginally lower scores. Conversely, patients operated under general anaesthesia present distinct patterns. LGG patients display more variable temporal trends, with complete recovery in the late-FU period. However, HGGs demonstrate a slight deterioration that persists over time, with the notable exception of the clock drawing test, in which full recovery was observed. These findings are further supported by the corresponding graph: even when considering a larger sample, as nearly all patients underwent the clock drawing test compared to the other two tests, the results remain consistent. Figure 4 shows the trend of the mean value of the clock drawing test across the three phases of the study, illustrating different trends according to the type of surgery (awake or asleep). Except for minor variability in baseline mean values, a uniform trajectory emerged across all cases, characterised by slight deterioration in the early postoperative phase, followed by improvement and culminating in complete recovery at late FU.

### 3.3. BPI Based on nTMS Data

The brain plasticity index was calculated only for 54 patients according to our clinical imaging pipeline criteria, since the comparison between pre- and postoperative nTMS maps was based on the anatomical regions’ alignment. We analysed the quantitative and qualitative data and graphically represented the maps comparing pre- and postoperative results, showing the projections on the right (Rh) and left (Lh) hemispheres.

#### 3.3.1. Quantitative Analysis

**Comparison between LGGs and HGGs**

We illustrated quantitative displacement patterns for motor function and for three cognitive domains: language, calculation, and visuo-spatial. The figures compare displacement across both hemispheres for all lesions and separately for LGGs versus HGGs. The displacement graphs display a colour scale ranging from yellow to purple, which indicates the proximity of the affected area to the displaced area. Purple denotes areas that are closer, whilst yellow represents areas that are further away. Unfortunately, we calculated *p* values ≥ 0.05 for all data we analysed, and therefore no statistically significant results were obtained. Nevertheless, preliminary results are explained in the following paragraph.

***Motor:*** Quantitative analysis across different hemispheres and the whole brain considering all lesions using box plots to demonstrate the distribution of BPI values: mean displacements of 21.08 mm in the Lh, 50.84 mm in the Rh, and 29.44 mm for the whole brain. Figure 5.1 (HGGs) box plots demonstrate the distribution of BPI values, with mean displacement of 21.08 mm in the Lh, 63.60 mm in the Rh, and 28.51 mm for the whole brain. Figure 5.2 (LGGs) shows the mean displacement of 19.58 mm in the Lh, 38.80 mm in the Rh, and 36.95 mm for the whole brain.

***Language:*** Quantitative analysis revealed the distribution of BPI values, with mean displacement of 34.71 mm in the Lh, 77.91 mm in the Rh, and 49.71 mm for the whole brain. Figure 6.1 (HGGs) box plots demonstrate the distribution of plasticity index values, with mean displacement of 59.33 mm in the Lh, 54.04 mm in the Rh, and 56.68 mm for the whole brain. Figure 6.2 (LGGs) shows mean displacement of 29.25 mm in the Lh, 79.32 mm in the Rh, and 30.89 mm for the whole brain.

***Calculation:*** Quantitative analysis reveals the distribution of BPI values, with mean displacement of 72.11 mm in the Lh, 47.52 mm in the Rh, and 55.14 mm for the whole brain. In Figure 7.1 (quantitative displacement of the calculation function in HGGs), box plots show mean displacement of 72.72 mm in the Lh, 48.11 mm in the Rh, and 65.69 mm for the whole brain. Figure 7.2 For LGGs mean displacement of 50.42 mm in the Lh, 41.29 mm in the Rh, and 47.52 mm for the whole brain.

**Figure 8:**
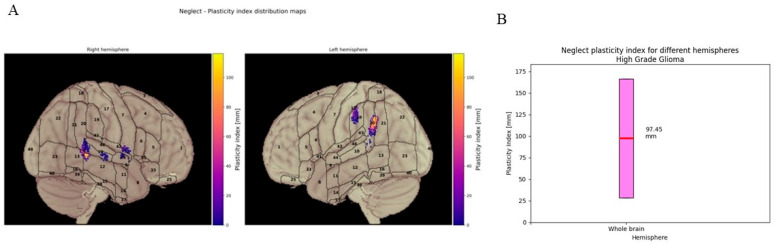
Visuo-spatial function brain plasticity index. Quantitative displacement of the visuo-spatial function in high-grade gliomas. (**A**) Visuo-spatial plasticity index distribution maps displaying the spatial pattern of cortical reorganisation across both hemispheres. Graphic representation illustrates the degree displacement with color-coded intensity values ranging from purple to yellow. (**B**) Quantitative analysis of visuo-spatial plasticity index for the whole brain. Box plots demonstrate the distribution of plasticity index values, with mean displacement of 97.45 mm.

***Visuo-spatial functions:*** Due to insufficient data, quantitative displacement of VSf for LGGs could not be measured, only that for HGGs, for which average displacement up to 97 mm was obtained. See Figure 8.

#### 3.3.2. Qualitative Analysis

Given that displacement metrics (in mm) and their averages provided a quantitative measure, our aim was to derive a qualitative characterisation of the anatomical regions involved in functional reorganisation, namely to determine where functions relocate based on cortical parcellation schemes (cps).

**Figure 9:**
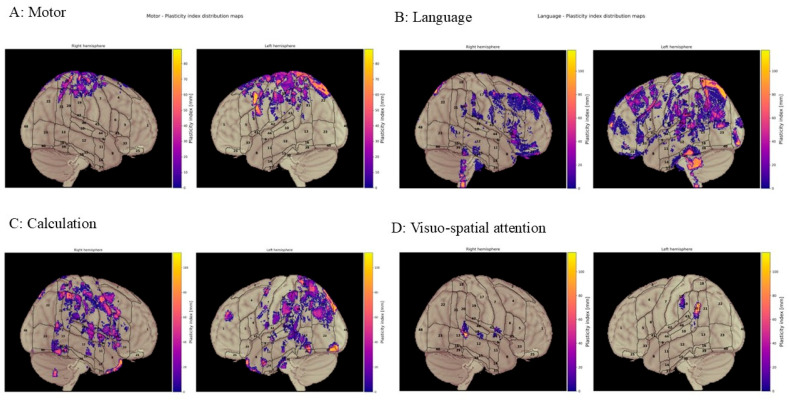
Representation of the final cumulative qualitative displacement for motor (**A**), language (**B**), calculation (**C**), and visuo-spatial attention (**D**) comparing the right and left hemispheres, including all oncological patients (Harvard–Oxford cortical parcellation system).

For language, according to cps-based anatomical classification using the Harvard–Oxford Atlas, left hemisphere (Lh) reorganisation showed displacement from the central opercular cortex toward the pars opercularis of the inferior frontal gyrus (IFG), the supramarginal gyrus (SMG) of the parietal cortex, and the temporal planum. In the right hemisphere (Rh), displacement patterns included shifts from the SMG and central opercular cortex to the frontal operculum, from the parietal operculum to the frontal operculum, from the temporal planum to the pars opercularis of the IFG, and from the temporal planum to the SMG and angular gyrus (AG) of the parietal cortex (Figure 9).

We extended the qualitative displacement analysis to encompass additional cognitive functions. Regarding calculation, involvement of the frontoparietal circuit was observed. For motor function, reorganisation occurred around the central sulcus: pre-centrally, corresponding to the primary motor cortex (M1, area 4) and premotor area (PM), and post-centrally, meaning the primary somatosensory cortex (S1). Concerning visuo-spatial function, the available data proved insufficient to determine a definitive qualitative displacement.

#### 3.3.3. Relationship Between BPI and MMSE

**Figure 10:**
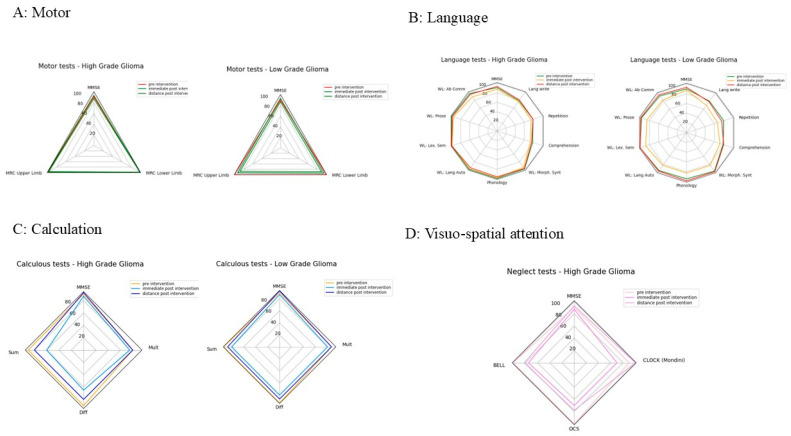
Clinical results and correlation with BPI, distinguishing between HGGs and LGGs for specific functions. (**A**) Spider plot representation of motor area displacement (MRC upper limb and MRC lower limb) compared to the variation in MMSE mean values during the three phases of the study. Data include HGGs (**left**) and LGGs (**right**). The triangle shape shows motor performance. If the shape expands outward after intervention → improvement. (**B**) Spider plot representation of the language functions displacement for HGGs and LGGs (Lang write, Repetition, Comprehension WL: Morph. Synt, Phonology, WL: Lang Auto, WL: Lex. Sem, WL: Prose, WL: Ab Comm) compared to the variation in MMSE mean values during the three phases of the study. A larger, more circular shape indicates better overall language function. We can compare how different language domains change over time. (**C**) Spider plot representation of calculation function displacement (Mult, Diff, Sum) compared to the variation in MMSE mean values during the three phases of the study. Data include HGGs and LGGs. Diamond-like shapes show strengths/weaknesses in arithmetic skills. (**D**) Spider plot representation of visuo-spatial function displacement (CLOCK (Mondini), OCS, Bell) compared to the variation in MMSE mean values during the three phases of the study. Data include only HGGs. A larger area indicates better attention/visuo-spatial ability.

Finally, we evaluated the clinical results and the average functional recovery immediately after surgery and during the FU period to evaluate any correlation between BPI and the age of education/intellectual status of each patient, distinguishing LGGs from HGGs. We searched for a correlation between the final performance on the AAT and histology. We collected clinical and neuropsychological assessments with language, summing the results and correlating with BPI for each patient. Then, we correlated the previous results with the MMSE. First, we evaluated the correlation between the estimated BPI and the variation in MMSE test values (expressed in percentages, considering the maximum value of the test). Only for Lh were results counted (because of not enough data on Rh). For HGG patients, there was a good correlation between BPI and MMSE variation (R = −0.80): the higher the BPI, the more difficult cognitive recovery was (R = −0,80). Coherently, for LGGs the trend was the same, although the correlation was much lower (R = −0.17). No significant data for VSf were obtained after our analysis, due to a limited number of cases and for the frame of our clinical/imaging pipeline. For HGGs, an average of displacement up to 97 mm was obtained. Figure 10 presents the clinical results and their correlation with BPI, indicating that extensive reorganisation reflects greater network disruption, not necessarily better compensation.

## 4. Discussion

This work proposes the synergistic use of complementary non-invasive instruments to optimise surgical outcomes for LGG/HGG patients and to provide advanced mapping capabilities, establishing a predictive model for the functional reshaping of brain circuits and ensuring the highest QoL, enabling patients to enjoy a normal life after surgery.

Analysing BPI through the non-invasive method of nTMS represents a beneficial strategy to study and predict the functional reorganisation of cortical circuits [1,14,25,45] The main purpose of integrating three fundamental pillars, such as tumour classification (LGGs/HGGs), mapping and surgical techniques, and understanding of brain plasticity, is to examine how glioma grade influences cognitive network reorganisation. We compared Ln, C, VSf between patients with LGG/HGGs operated on awake/asleep to elucidate how BPI may be affected. Furthermore, we analysed the relationship between BPI and postoperative cognitive assessments.

We know that LGGs and HGGs have different patterns of growth and consequently exert markedly different influences on large-scale brain networks, both in terms of the extent and mechanisms of plastic reorganisation, even before any surgical intervention. To address this, we illustrate a comparison of network maps stratified by timing (preoperative vs. postoperative).

Specifically, we separated analyses of LGG and HGG cohorts to highlight differential presurgical network adaptations and postoperative reconfiguration, distinguishing the quantitative and qualitative types of analyses. Moreover, we quantified and visualised pre- vs. postoperative changes in connectivity measures, emphasising how slow-growing LGGs promote gradual compensatory remodelling while HGGs precipitate more abrupt, limited plastic responses. This modification strengthens the argument that the observed network alterations and recovery patterns cannot be interpreted without considering both tumour grade and timepoint (pre- vs. postsurgery). We demonstrated the following.

- Plasticity is function-specific: Motor networks are more constrained, whereas language and calculation are highly plastic.
- Contralateral hemisphere compensation (especially for LGGs): Language (normally left-lateralised) shifts toward the Rh. Calculation shows stronger Lh plasticity. This supports the idea that less dominant representations provide reserve capacity.
- LGGs vs. HGGs: HGGs induce greater functional displacement, but poorer recovery, whereas LGGs, due to slower growth, allow more effective adaptation.
- Awake surgery remains the gold standard despite transient early deficits, awake surgery yields better long-term preservation, especially in HGGs and in visuo-spatial functions.
- nTMS improves preoperative planning and may reduce the need for awake surgery in selected cases.
- Connectome perspective: Cortical plasticity is high, but white-matter tracts are the real surgical limit.
- nTMS + DTI + DCS (or CCEPs) provides a network-based safety framework.

We based our methodology on an extended use of nTMS, enabling preoperative mapping of cognitive networks and assessment of neuroplastic reorganisation in response to tumour growth and surgery. Indeed, nTMS not only enables the identification of eloquent cortical areas, but also provides a window into the dynamic process of neuroplastic adaptation in response to glioma growth/surgical event.

Unexpectedly, we did not find any correlation between the brain plasticity index and tumour volume (linear regression for left and right hemisphere for LGGs and for HGGs below, Figure 1 and Figure 2): LGGs in the left hemisphere (R = 0.28) and right hemisphere (R = 1.00); HGGs in the left hemisphere (R = 0.31) and right hemisphere (R = 1.00).

Our preliminary data suggest that tumour volume does not significantly alter plasticity mapping, since this relationship has not yet achieved statistical significance. Although highly probable based on our observations, this hypothesis requires further validation with larger cohorts and more extensive follow-up data. Understanding the precise relationship between tumour characteristics—particularly glioma grade—and plasticity metrics remains a critical objective for establishing a robust predictive model.

We can clarify that no nTMS is performed immediately after surgery, when we normally limit our evaluation to a clinical and cognitive evaluation to understand the type of cognitive impairment (if present) and the consequent rehabilitation pathway. The functional reorganisation is evaluated based on clinical-cognitive and nTMS analyses after approximately 3 months from surgery, to minimise all possible biases (surgical artefacts, mass removal, oedema, patient fatigue after surgery reducing compliance during nTMS, and cognitive evaluation with the speech therapist or neuropsychologist).

### The Whole BPI: Considerations for Each Specific Function

For motor function, the whole displacement has an average of ~30 mm, whereas for language it is ~50 mm and for calculation even up to ~55 mm. Therefore, C seems to be most displaced and with linguistic pathways the more plastic ones. Concerning the comparison between BPI obtained in Rh/Lh, we had a major displacement of motor function in the Rh (51 mm), whit a limited displacement in the Lh (22 mm). For language, again, the major displacement is in the Rh compared to the Lh (77 vs. 34 mm). Finally, for C the major displacement has been obtained in the Lh (72 mm vs. 48 mm).

The major BPI has been obtained in the Rh for motor and language functions, conversely in the Lh for calculation. Considering that normally language is lateralised in the Lh, whereas the geometric- arithmetic circuits have a predominance in the Rh [46,47], our results could be interpreted as a major involvement of the contralateral hemisphere. Plastic reshaping is quantitatively higher where the specific function has a minor representation. Therefore, functional highlighting in the Rh for language and in the Lh for calculation could suggest that the contralateral hemisphere works to compensate for any impairment of the cognitive circuit into consideration. Finally, concerning motor function, the BPI is more limited across Lh/Rh along the areas of the central/precentral and post-central cortical regions.

For motor function, the whole displacement for motor function had an average of about 30 mm (for language ~50 mm and for calculation ~55 mm). Motor function had major displacement on the right hemisphere (51 mm) and more limited on the left (21 mm), with overall displacement more restrained than that of other functions, suggesting a modulation around the anatomical areas of motor/premotor cortex/primary sensitive motor area. For HGGs and LGGs, the major motor function displacement was on the right (more pronounced in HGGs than in LGGs: 64 mm vs. 39 mm), with more restrained displacement on the left hemisphere (LGGs 20 mm vs. HGGs 21 mm), comparable between the two types. Finally, the overall results were higher for LGGs than for HGGs (37 mm vs. 29 mm).

For the language function, the displacement reached ~50 mm, with larger displacement in the Rh. Overall displacement was higher for HGGs than for LGGs (57 mm vs. 31 mm). However, surprisingly for HGGs in the Lh, displacement was more significant (60 mm), whereas for LGGs it was major in the Rh (~80 mm). Consistent with what was previously hypothesised, this could suggest a more relevant role of the Rh for LGGs in the activation of a functional compensatory mechanism that could be helpful to mitigate the cognitive impairment due to the presence of the lesion.

The overall BPI for the calculation function is more represented on the left than on the right hemisphere, considering our cumulative results (average displacement on the whole brain 55.14 mm). Overall displacement for arithmetic function was more evident in the Lh for LGGs and HGGs but was more significant for HGGs (72 mm Lh vs. 48.11 mm Rh average displacement). A possible interpretation could be the major active role of the Lh in the LGG patients since displacement in the Rh was lower than in the Lh (41 vs. 50 mm), suggesting that in functional reshaping, the Lh plays a major role in LGG patients. This could be motivated by the slower rate of growth of LGGs and more adaptive time to activate compensatory mechanisms.

For VSf, no significant data were obtained after our analysis due to the limited number of cases and for the frame of our clinical/imaging pipeline. For HGGs, average displacement up to 97 mm was obtained.

Complexity of plasticity and functional recovery: In HGGs, a robust negative correlation existed between higher BPI values and increased difficulty in functional recovery (R = −0.80). Elevated BPI scores were associated with poorer cognitive performance on the MMSE. Whilst LGGs similarly demonstrated that higher BPI correlates with greater recovery challenges, this negative correlation was considerably weaker (R = −0.17). This disparity may be attributed to the slower growth rate of LGGs, which potentially affords the brain greater temporal capacity to activate compensatory mechanisms.

Language and motor function represented the most frequently mapped domains during awake brain surgery. Language remains the most extensively tested cognitive domain; however, a positive trend towards the implementation of a broader range of cognitive tests is observed [48,49,50]. This is essential to benefit from a high QoL after surgery, enjoying normal life and recovering professional/social abilities [8]. The evaluation of subcortical tracts involved in cognitive networks through tractographies derived from both anatomical tensors and nTMS data further enhances the understanding of functional connectivity and guides safer resection strategies [23,24,37,51]. Nowadays, innovative tools for functional surgery under general anaesthesia have emerged: CCEPs [52,53] have been integrated into asleep procedures to monitor arcuate fasciculus (AF) functional integrity and represent a modern innovation that could be implemented in the surgical setting with awake validation [54].

Based on the comparative analysis of mean scores between patients who underwent surgery under general anaesthesia and awake surgery, a notable pattern emerges. Although awake surgery is associated with a decline in performance during the early postoperative period, likely attributable to the effects of DCS, long-term outcomes demonstrate that awake surgery yields superior results across nearly all subfunctions, with a clear improvement observed in cognitive test performances. The trajectory of language functions throughout the FU period reveals several important patterns (Figure 2). In the preoperative phase, there is no significant difference in mean scores between patients, with slightly lower values recorded for patients with LGGs. However, in the first (post+) FU phase, a decline in cognitive scores becomes evident in patients operated on awake, particularly among LGGs. Subsequently, in the second (post++) FU phase, a marked improvement in cognitive test performance is observed, with comparable values between LGGs and HGGs operated under both awake and asleep conditions, with superior values noted for LGGs. Late FU assessments reveal nearly overlapping values when comparing awake and asleep procedures for both LGG/HGGs. These findings suggest that nTMS mapping represents a valid strategy for mitigating postoperative cognitive deficits, enabling substantially equivalent functional recovery irrespective of the surgical modality employed, when utilised as a preoperative planning tool. nTMS has emerged as a reliable method for preoperative language mapping, optimising intraoperative DCS and potentially enhancing tumour resection whilst preserving function [21,26,37,54,55,56,57]. The ability to identify non-eloquent brain regions preoperatively facilitates the delineation of safe resection zones, with the potential to operate on selected patients without recourse to awake surgery.

Examining changes in cognitive function across three arithmetic tasks—addition (SOM), subtraction (DIF), and multiplication (MOLT)—throughout the three phases of the study reveals distinct patterns between surgical approaches (Figure 3). In the preoperative phase, there is not so much of a difference between LGGs and HGGs, but rather between awake and asleep surgeries, with consistently lower mean values observed in patients operated on under general anaesthesia. In the postoperative phase, a divergent trend emerges patients who underwent awake surgery demonstrate a moderate decline in performance, particularly evident in the HGG group, followed by near-complete recovery during late FU. Conversely, patients operated upon under general anaesthesia maintain lower mean scores throughout the observation period, with only minimal improvements over time. The sole exception is represented by the MOLT (multiplication) function, which shows immediate postoperative improvement.

Overall, these findings could be attributable to the effects of DCS during awake procedures, with a transitory alteration in functional circuits, whose postoperative clinical impact may result in a slight impairment of specific cognitive functions. The late results during the FU period underscore the greater recovery potential associated with awake surgery, particularly in the long term. This disparity is especially pronounced within the HGG cohort: patients who underwent general anaesthesia exhibit persistently lower mean values, whilst those operated upon with awake monitoring during surgery achieve near-complete recovery. However, we noted that both groups maintained values comparable to their preoperative baseline during late FU, albeit with a slight decrease. This raises the question of whether patient selection criteria may influence these outcomes, and whether alternative candidates for awake surgery might demonstrate different recovery patterns.

The comparison of VSf and neglect assessment tests—Bell test, Broken Hearts (OCS), and clock drawing test (CLOCK Mondini)—evaluated across the three phases of the study demonstrates distinct trajectories by surgery type (Figure 4). Patients selected for awake surgery demonstrate the poorest outcomes in the Bell test and clock drawing test during the early postoperative phase. However, all patients ultimately achieve complete recovery. Conversely, the mean values of patients operated under general anaesthesia vary according to tumour grade: LGGs demonstrate favourable outcomes, with complete recovery achieved after ~1 month FU. The sole exception is represented by the clock drawing test, where complete recovery is observed in all cases, regardless of tumour grade or surgical approach.

Within the HGG cohort, patients who underwent awake surgery exhibit complete recovery across all three visuo-spatial tests, whilst those operated on under general anaesthesia present persistently poorer outcomes, except for the clock drawing test, where complete recovery is observed in all cases. In cases where neglect deficits arise, typically resulting from damage to the right hemisphere due to the presence of a HGGs, the awake approach appears to be the optimal strategy for maximising cognitive preservation.

Our results corroborate these observations and align with established evidence in the literature, confirming that awake surgery yields superior functional preservation and recovery trajectories. The transient functional decline associated with awake surgery is likely due to the resection, which can be safely pushed closer to eloquent cortical areas and subcortical tracts, thereby inducing temporary functional disruption that does not preclude subsequent recovery, as previously demonstrated [44].

Neuroplasticity, described as the ‘fourth dimension’ of neurosurgery [58], represents the brain’s intrinsic capacity to dynamically redistribute neural networks over time, protecting critical functional regions from tumoural damage. However, this adaptive capacity exhibits marked hierarchical organisation throughout cerebral architecture. As Duffau highlights [15], the cerebral cortex possesses considerable plastic potential, albeit constrained by the ‘minimal common brain’—those essential regions and connections that cannot be redistributed without compromising vital functions. In contrast, deep white-matter tracts demonstrate substantially inferior reorganisation capacity. This stratified plasticity pattern has profound implications for surgical planning: cortical lesions or those involving superficial white matter may benefit from compensatory mechanisms over time, whereas deep structure involvement demands rigorous preservation. Indeed, in glioma resection planning, the true surgical limitation is not tumour extension, but rather the preservation of deep white-matter tracts, whereas the cortical surface can be mapped and non-invasively stimulated. These pathways constitute the fundamental architecture maintaining cerebral network connectivity and represent the critical boundaries of the human connectome. DCS represents the gold standard for intraoperative assessment, as white-matter connectivity is fundamental for preservation of complex cognitive skills. Nowadays, CCEPs represent a valid alternative to monitor the integrity of the white-matter bundles during asleep surgery. The dynamic interaction among multiple neural networks operating conjointly, unconsciously generating appropriate responses to environmental stimuli, represents the most effective concept for balancing oncological radicality with preservation of cognitive capabilities [59].

Interindividual variability in functional localisation renders real-time intraoperative monitoring fundamentally important, providing precise anatomical-functional correlation. The integration of preoperative mapping techniques—particularly nTMS and DTI tractography—with intraoperative DCS monitoring is indispensable for safe, functionally oriented surgical planning that respects both the plastic potential of cortical regions and the rigid constraints imposed by deep white-matter architecture [58].

Future prospects:

- Test–retest reliability: The importance of assessing non-invasively BPI over repeated measurements. Further data are needed to test the reproducibility of BPI across sessions.
- Sensitivity to technical variations in nTMS coil positioning: Coil positioning may influence measurements. The standardisation of coil placement is essential, and its variability could affect BPI values, so potential mitigation strategies must be found.
- Threshold for clinically significant BPI values: Given the exploratory nature of this study, we have refrained from defining a strict clinical cut-off at this stage. However, we need to work on possible approaches for determining clinically meaningful thresholds in future studies, including larger cohort validation and outcome-based calibration.

Limitations: Our study did not include a control group of healthy subjects for normative BPI values, which could be useful to determine if the brain plasticity index we obtained is due to a functional reshaping concerted to the presence/removal of a lesion or present per se. Moreover, the tumour volume–plasticity relationship has not been statistically validated yet. Limited data on visuo-spatial plasticity are available in our case series.

BPI was calculated for only 54 patients, who were then further subdivided by tumour grade, hemisphere, and cognitive domain, resulting in very small subgroups. This severely limits the generalizability of our findings, which need to be validated with greater case series and further stratification based on tumour location, functional network which were involved, level of scholarship and cognitive reserve.

The awake vs. asleep surgical groups were neither randomised nor balanced for baseline characteristics such as tumour location, volume, and grade. Therefore, a direct comparison without correction for confounders has very limited validity. Moreover, oncological outcome parameters—primarily extent of resection (EOR), and secondarily overall survival (OS) and progression-free survival (PFS)—must be considered when assessing whether nTMS-guided asleep surgery is truly comparable to awake surgery. Further data are needed to validate these preliminary findings.

A longer follow-up period is necessary to better understand genuine brain reorganisation, less influenced by the acute effects of surgery.

To sum up, to the best of our knowledge, this is the first study to use a multiparametric paradigm to non-invasively acquire data on cortical functional reshaping in oncological patients, studying cognitive performance and stratifying results based on histological classification (HGGs and LGGs) and type of surgery (awake vs. asleep).

## 5. Conclusions

We propose a non-invasive, multimodal method for assessing brain plasticity across different cognitive functions in glioma patients. The brain plasticity index captures function-specific and hemispheric reorganisation, differentiating between LGGs and HGGs and asleep and awake procedures. Our multimodal approach shows a promising association between BPI and recovery patterns of oncological patients, with prospective validation required to establish true predictive capacity.

## Figures and Tables

**Figure 5:**
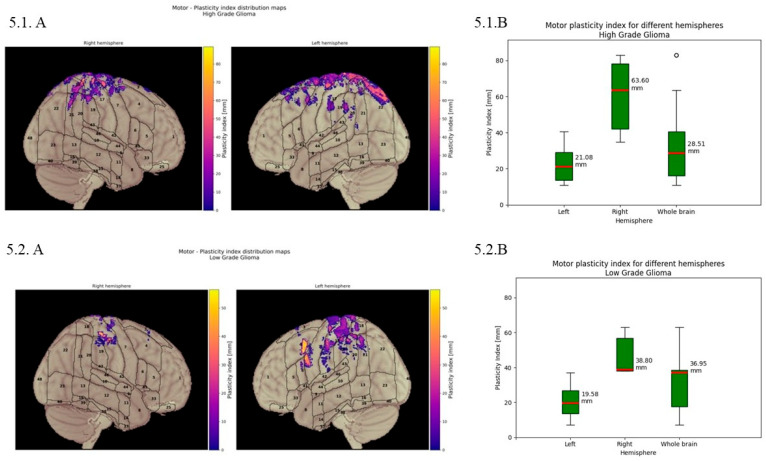
Motor brain plasticity index. (**5.1**) Quantitative displacement of the motor function in HGGs. (**A**) Motor plasticity index distribution maps displaying the spatial pattern of cortical reorganisation across both hemispheres. Graphic representation illustrates the degree of displacement with color-coded intensity values ranging from purple to yellow. (**B**) Quantitative analysis of motor plasticity index for different hemispheres. Box plots demonstrate the distribution of plasticity index values, with a mean displacement of 21.08 mm in the left hemisphere, 63.60 mm in the right hemisphere, and 28.51 mm for the whole brain. (**5.2**) Quantitative displacement of the motor function in LGGs. (**A**) Motor plasticity index distribution maps displaying the spatial pattern of cortical reorganisation across both hemispheres. Graphic representation illustrates the degree displacement with color-coded intensity values ranging from purple to yellow. (**B**) Quantitative analysis of motor plasticity index for different hemispheres. Box plots demonstrate the distribution of plasticity index values, with mean displacement of 19.58 mm in the left hemisphere, 38.80 mm in the right hemisphere, and 36.95 mm for the whole brain.

**Figure 6:**
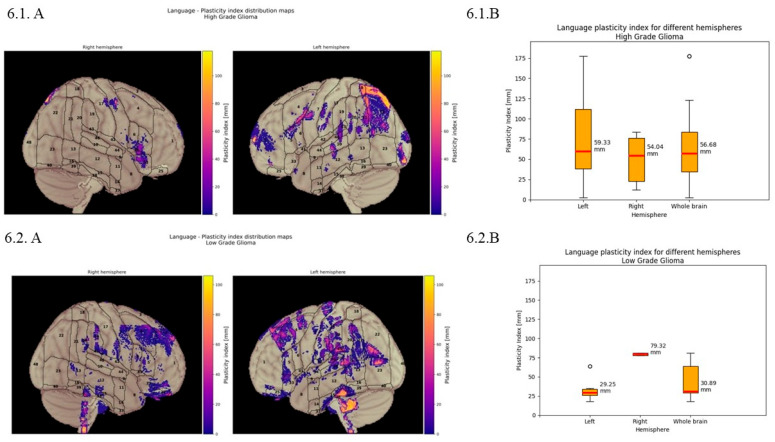
Language brain plasticity index. (**6.1**) Quantitative displacement of the language function in HGGs. (**A**) Language plasticity index distribution maps displaying the spatial pattern of cortical reorganisation across both hemispheres. Graphic representation illustrates the degree of displacement with color-coded intensity values ranging from purple to yellow. (**B**) Quantitative analysis of the language plasticity index for different hemispheres. Box plots demonstrate the distribution of plasticity index values, with mean displacement of 59.33 mm in the left hemisphere, 54.04 mm in the right hemisphere, and 56.68 mm for the whole brain. (**6.2**) Quantitative displacement of the language function in LGGs. (**A**) Language plasticity index distribution maps displaying the spatial pattern of cortical reorganisation across both hemispheres. Graphic representation illustrates the degree displacement with color-coded intensity values ranging from purple to yellow. (**B**) Quantitative analysis of language plasticity index for different hemispheres. Box plots demonstrate the distribution of plasticity index values, with mean displacement of 29.25 mm in the left hemisphere, 79.32 mm in the right hemisphere, and 30.89 mm across the whole brain.

**Figure 7:**
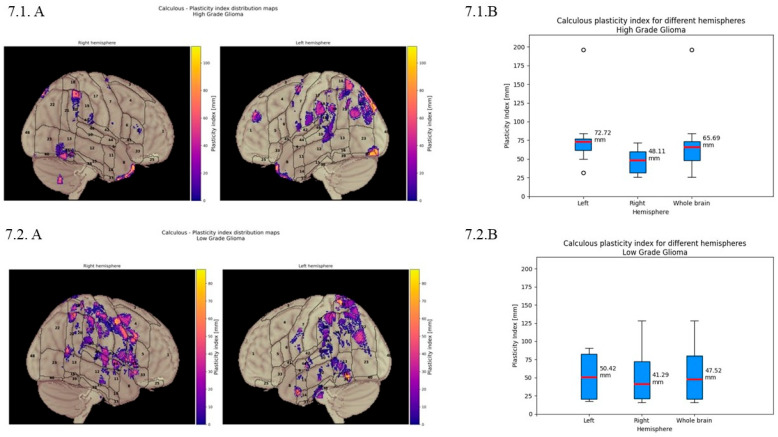
Calculation brain plasticity index. (**7.1**) Quantitative displacement of the calculation function in HGGs. (**A**) Calculation of plasticity index distribution maps displaying the spatial pattern of cortical reorganisation across both hemispheres. Graphic representation illustrates the degree of displacement with color-coded intensity values ranging from purple to yellow. (**B**) Quantitative analysis of calculation plasticity index for different hemispheres. Box plots demonstrate the distribution of plasticity index values, with a mean displacement of 72.72 mm in the left hemisphere, 48.11 mm in the right hemisphere, and 65.69 mm for the whole brain. (**7.2**) Quantitative displacement of the calculation function in LGGs. (**A**) calculation plasticity index distribution maps displaying the spatial pattern of cortical reorganisation across both hemispheres. Graphic representation illustrates the degree displacement with color-coded intensity values ranging from purple to yellow. (**B**) Quantitative analysis of calculation plasticity index for different hemispheres. Box plots demonstrate the distribution of plasticity index values, with mean displacement of 50.42 mm in the left hemisphere, 41.29 mm in the right hemisphere, and 47.52 mm across the whole brain.

**Table 1:** Summary of glioma patients analysed.

P	Lobe (F, T, P, O)	Site (L/R)	Grade WHO 2021	IDH (0 = WT/1 = Mutated)	Histology WHO 2021	Age (Surgery)	Education (Years)
1	T	L	2	1	Astrocytoma	23	13
2	T	L	3	1	Astrocytoma	31	15
4	F	R	2	1	Astrocytoma	30	16
5	F	L	4	0	GBM	60	10
8	T	R	4	0	GBM	55	13
9	F	L	4	0	GBM	72	18
10	P	L	3	1	Astrocytoma with focal area of anaplastic progression	32	8
11	T	L	4	0	GBM	58	8
12	T	R	3	1	Astrocytoma	30	17
13	F	R	3	1	Astrocytoma	28	8
14	F–T	R	4	1	Astrocytoma	49	8
15	F	R	2	1	Oligodendroglioma	61	13
16	P	L	4	0	GBM	68	13
17	Splenium CC		3	1	Astrocytoma	47	13
18	T	L	4	0	GBM	46	13
19	F–T–In		2	1	Oligodendroglioma	67	13
20	F	L	4	0	GBM	54	13
21	F	R	3	1	Astrocytoma	55	13
23	T	R	2	1	Oligodendroglioma	37	13
24	F	L	2	1	Oligodendroglioma	53	12
25	P	L	3	1	Astrocytoma	42	17
27	T	R	4	0	GBM	57	18
28	F	R	2	1	Astrocytoma	28	13
29	F	L	2	1	Oligodendroglioma	44	16
30	F–I	R	4	0	GBM	54	8
31	T–I	L	2	1	Astrocytoma	43	8
32	T–I	L	2	1	Oligodendroglioma	31	9
33	F	L	4	0	GBM	44	13
35	F–I	R	4	0	Gliosarcoma	61	13
36	T	L	2	1	Oligodendroglioma	39	13
37	P	L	2	1	Astrocytoma	49	10
38	P	L	4	0	Gliosarcoma	47	13
39	P	R	2	1	Glioma	28	17
40	F	R	2	1	Astrocytoma	40	14
41	F	R	2	1	Astrocytoma	42	14
45	F	R	3	1	Astrocytoma	22	10
47	T	R	4	0	GBM	43	13
48	F	L	2	1	Astrocytoma	35	13
50	P	R	4	0	GBM	59	8
52	F	R	4	0	GBM	65	10
54	F–I	R	2	1	Oligodendroglioma	34	13
56	F	L	4	0	GBM	63	8
59	T	R	4	0	GBM	72	13
60	F	R	4	0	GBM	69	8
61	F	L/R	4	1	Oligodendroglioma	40	8
65	T	L	2	0	Oligodendroglioma	76	16
66	F	L	2	1	Astrocytoma	32	11
67	F	L	4	1	Astrocytoma	39	13
68	F	R	3	0	Oligodendroglioma	62	13
69	F–T–I	L	2	1	Astrocytoma	32	12
71	F	L	1	0	Subependymal giant-cell astrocytoma (SEGA)	20	13

F = frontal, T = temporal. I = insular, P = parietal, L = left, R = right, GBM = glioblastoma, IDH 0 = wild type, 1 = mutant.

## Data Availability

Data collected for this work are unavailable due to privacy or ethical restrictions imposed by the Ethics Committee of the Area Vasta Centro, University Hospital of Careggi (Florence, Italy). If necessary, based on region, the authors can share the pipeline script and the anonymised dataset for clinical/scientific purposes.

## Supplementary Materials

The following supporting information can be downloaded at https://www.mdpi.com/article/10.3390/cancers18091405/s1.
