# Supplementary material for: Non-Invasive Measurement of Cortical Plasticity in Brain Tumour Surgery: A Monocentric Experience of nTMS Mapping and Definition of Cognitive Reshaping Based on Tumour Histological Grade
Source: Cancers (Basel). 2026 Apr 28;18(9):1405. doi: 10.3390/cancers18091405 (PMC13162762; doi:10.3390/cancers18091405)
Supplement: Supplementary file 1 [file cancers-18-01405-s001.zip › cancers-4159172-supplementary.pdf]

Table Language *[Organisation of cognitive test data entry for each patient (P). In particular, the values relating to the tests obtained before the operation can be seen. Similarly, the post-operative data has been organised].*

	A	B	C	D	E	F	G	H	I	J	K	L	M	N
1	P	Functions	Emis	MMSE	LS:Ab Com	LS:ArtPros	LS:Les Sem	LS:L Auto	LS:Fono	LS:MorfSint	DEN	COM	RIP	Ling scr
2	1	M/L/C	Bilat	25,59	4	5	5	4	5	5	3	3	4	4
3	2	M/L no*/C	Bilat	25,75	4	5	5	5	5	5	4	4	4	4
4	3	M/L(*bilator	Bilat	14,59	4	4	4	4	4	4	4	4	4	4
5	4	M*no post/L	Bilat	28,1	5	5	5	5	5	5	4	4	4	4
6	5	M/L	Left	29,49	5	5	5	5	5	5	4	4	4	4
7	6	M/L/C*no p	Right	29,99	5	5	5	5	5	5	4	4	4	4
8	7	M* notdone	Bilat	28,85	5	5	5	5	5	5	4	4	4	4
9	8	M/L/C	Right	29,99	5	5	5	5	5	5	4	4	4	4
10	9	M*no post/l	Left	27,5	5	5	5	5	5	5	4	4	4	4
11	10	M/L/C*no p	Left	27,75	5	5	5	5	5	5	4	4	4	4
12	11	M (*R)/L	Left	25,97	5	5	5	5	5	5	4	4	4	4
13	12	M/L/C (*onl	Left	28,1	5	5	5	5	5	5	4	4	4	4
14	13	M/L/C	Bilat	28,19	5	5	5	5	5	5	4	4	4	4
15	14	M/L/C	Bilat	29,62	5	5	5	5	5	5	4	4	4	4
16	15	M/L/N/C*Nc	Right	25,49	4	5	5	5	5	5	4	4	4	4
17	16	M/L/C/N *N	Left	25,49	5	5	5	5	5	5	4	4	4	4
18	17	M/L/C	Bilat	28,89	5	5	5	5	5	5	4	4	4	4
19	18	M/L	Left	27,89	5	5	5	5	5	5	4	4	4	4
20	19	M *No pre c	Left	28,49	5	5	5	5	5	5	4	4	4	4
21	20	M/L	Bilat	28,31	5	5	5	5	5	5	4	4	4	4
22	21	M/L/C	Bilat	28,31	5	5	5	5	5	5	4	4	4	4
23	22	M/L/N/C*Nc	Bilat	26,59	5	5	5	5	5	5	4	4	3	4
24	23	M/L/C	Bilat	28,75	5	5	5	5	5	5	4	4	4	4
25	24	M/L/C*notp	Bilat	28,99	4	5	5	5	4	5	4	4	4	4
26	25	M/L/C/N	Bilat	28,21	5	5	5	5	5	4	4	4	4	4
27	26	M/L/C*only	Bilat	23,99	4	5	5	5	5	5	4	4	3	3
28	27	M/L/C/N	Bilat	28,46	5	5	5	5	5	5	4	4	4	4
29	28	M/L/C	Bilat	27,59	5	5	5	5	5	5	4	4	4	4
30	29	M/L/C/N	Bilat	27,21	4	5	5	5	4	5	4	4	4	4
31	30	M/L/N/C*Nc	Bilat	20,97	4	5	5	5	5	5	4	4	4	4
32	31	M/L/C	Bilat	26,67	5	4	5	4	4	4	4	4	4	4
33	32	M*no postc	Bilat	16,75	4	4	5	4	4	4	4	4	4	4
34	33	M/L/C/N	Bilat	28,21	5	5	5	5	5	5	4	4	4	4
35	34	M/L/C	Left	27,21	4	4	5	4	4	4	4	3	3	4
36	35	M* notpost	Bilat	29,49	5	5	5	5	5	5	4	4	4	4
37	36	M/L/C	Left	28,75	5	5	5	5	5	5	4	4	4	4
38	37	M/L/C	Left	28,21	5	5	5	5	5	5	4	4	4	4
39	38	M/L/N/C*no	Bilat	17,89	4	5	5	4	4	4	3	2	3	2
40	39	L/C/N	Bilat	28,07	5	5	5	5	5	5	4	4	4	4
41	40	M/L/C	Bilat	28,21	5	5	5	5	5	5	4	4	4	4
42	41	M/L/C	Bilat	28,21	5	5	5	5	5	5	4	4	4	4
43	42	L	Bilat	24,99	5	4	5	5	5	5	4	4	3	4
44	43	M/L/C/N	Bilat	28,75	5	5	5	5	5	5	4	4	4	4
45	44	M/L/C/N(so	Bilat	28,89	5	5	5	5	5	5	4	4	4	4
46	45	M/L/C	Bilat	28,59	5	5	5	5	5	5	4	4	4	4
47	46	M/L/C	Bilat	29,19	5	5	5	5	5	5	4	4	4	4
48	47	M/L	Bilat	28,62	5	5	5	5	5	5	4	4	4	3
49	48	M/L/C/N	Bilat	28,75	5	5	5	5	5	5	4	4	4	4
50	49	M/L/C/N	Bilat	29,99	5	5	5	5	5	5	4	4	4	4
51	50	M/L/C/N	Bilat	29,97	5	5	5	5	5	5	4	4	4	4
52	51	M/L/C	Bilat	29,99	5	5	5	5	5	5	4	4	4	4
53	52	M/L/C	Bilat	28,46	5	5	5	5	5	5	4	4	4	4
54	53	M/L/C/N	Bilat	28,89	5	5	5	5	5	5	4	4	4	4
55	54	M/L/C/N	Bilat	28,75	5	5	5	5	5	5	4	4	4	4
56	55	M/L/C/N	Bilat	28,07	5	5	5	5	5	5	4	4	4	4
57	57	M/L/C/N	Bilat	27,1	5	5	5	5	5	5	4	4	4	4
58	58	M/L/C	Bilat	28,75	5	5	5	5	5	5	4	4	4	4
59	59	M/L/C/N	Bilat	29,86	5	5	5	5	5	5	4	4	4	4
60	60	M/L	Bilat	28,53	5	5	5	5	5	5	4	4	4	4
61	61	M/L/C	Bilat	26,38	5	5	5	5	5	5	4	4	4	4
62	62	M/L/C/N	Bilat	27,31	5	5	5	5	5	5	4	4	4	4
63	64	M/L/C/N	Bilat	27,21	5	5	5	5	5	5	4	4	4	4
64	65	M/L/C/N	Bilat	27,86	5	5	5	5	5	5	4	4	4	4
65	66	M/L	Bilat	24,75	5	5	5	5	5	5	4	4	4	4
66	67	M/L/C/N	Bilat	26,75	5	5	5	5	5	5	4	4	4	4
67	68	M/L/C	Bilat	28,49	5	5	5	5	5	5	4	4	4	4
68	69	M/L/C/N	Bilat	28,75	5	5	5	5	5	5	4	4	4	4
69	70	M/L/C/N	Bilat	28,07	5	5	5	5	5	5	4	4	4	4
70	71	M/L/C/N	Bilat	28,59	5	5	5	5	5	5	4	4	4	4

Table Calculations

	A	B	C	D	E	F	G
1	P	Functions	Emis	MMSE	SOM	DIF	MOLT
2	1	M/L/C	Bilat	25,59	1	1	1
3	2	M/L no*/C	Bilat	25,75	1	1	1
4	3	M/L(*bilator	Bilat	14,59	0	0	0
5	4	M*no post/L	Bilat	28,1	1	1	1
6	5	M/L	Left	29,49	1	1	1
7	6	M/L/C*no pc	Right	29,99	1	1	1
8	7	M* notdone	Bilat	28,85	1	1	1
9	8	M/L/C	Right	29,99	1	1	1
10	9	M*no post/L	Left	27,5	1	1	1
11	10	M/L/C*no pc	Left	27,75	1	1	0
12	11	M (*R)/L	Left	25,97	1	1	0
13	12	M/L/C (*only	Left	28,1	1	1	1
14	13	M/L/C	Bilat	28,19	1	1	1
15	14	M/L/C	Bilat	29,62	1	1	1
16	15	M/L/N/C*No	Right	25,49	1	1	1
17	16	M/L/C/N *Nc	Left	25,49	1	1	1
18	17	M/L/C	Bilat	28,89	1	1	1
19	18	M/L	Left	27,89	1	1	1
20	19	M *No pre o	Left	28,49	1	1	1
21	20	M/L	Bilat	28,31	1	1	1
22	21	M/L/C	Bilat	28,31	1	1	1
23	22	M/L/N/C*No	Bilat	26,59	1	1	1
24	23	M/L/C	Bilat	28,75	1	1	1
25	24	M/L/C*notp	Bilat	28,99	1	1	1
26	25	M/L/C/N	Bilat	28,21	1	1	1
27	26	M/L/C*only	Bilat	23,99	0	1	1
28	27	M/L/C/N	Bilat	28,46	1	1	1
29	28	M/L/C	Bilat	27,59	1	1	1
30	29	M/L/C/N	Bilat	27,21	1	1	1
31	30	M/L/N/C*No	Bilat	20,97	0	0	0
32	31	M/L/C	Bilat	26,67	0	0	0
33	32	M*no posto	Bilat	16,75	1	1	0
34	33	M/L/C/N	Bilat	28,21	1	1	1
35	34	M/L/C	Left	27,21	1	1	1
36	35	M* notpostc	Bilat	29,49	1	1	1
37	36	M/L/C	Left	28,75	1	1	1
38	37	M/L/C	Left	28,21	1	1	1
39	38	M/L/N/C*not	Bilat	17,89	0	0	0
40	39	L/C/N	Bilat	28,07	1	1	1
41	40	M/L/C	Bilat	28,21	1	1	1
42	41	M/L/C	Bilat	28,21	1	1	1
43	42	L	Bilat	24,99	1	1	1
44	43	M/L/C/N	Bilat	28,75	1	1	0
45	44	M/L/C/N(sol	Bilat	28,89	1	1	1
46	45	M/L/C	Bilat	28,59	0	1	1
47	46	M/L/C	Bilat	29,19	1	1	1
48	47	M/L	Bilat	28,62	1	0	0
49	48	M/L/C/N	Bilat	28,75	1	1	1
50	49	M/L/C/N	Bilat	29,99	0	1	1
51	50	M/L/C/N	Bilat	29,97	1	1	1
52	51	M/L/C	Bilat	29,99	1	1	1
53	52	M/L/C	Bilat	28,46	1	1	1
54	53	M/L/C/N	Bilat	28,89	1	1	1
55	54	M/L/C/N	Bilat	28,75	1	1	1
56	55	M/L/C/N	Bilat	28,07	1	1	1
57	57	M/L/C/N	Bilat	27,1	1	1	1
58	58	M/L/C	Bilat	28,75	1	1	1
59	59	M/L/C/N	Bilat	29,86	1	1	1
60	60	M/L	Bilat	28,53	1	0	1
61	61	M/L/C	Bilat	26,38	1	1	1
62	62	M/L/C/N	Bilat	27,31	1	1	1
63	64	M/L/C/N	Bilat	27,21	1	1	1
64	65	M/L/C/N	Bilat	27,86	1	1	1
65	66	M/L	Bilat	24,75	0	0	0
66	67	M/L/C/N	Bilat	26,75	1	1	1
67	68	M/L/C	Bilat	28,49	1	1	1
68	69	M/L/C/N	Bilat	28,75	1	1	1
69	70	M/L/C/N	Bilat	28,07	1	1	1
70	71	M/L/C/N	Bilat	28,59	1	1	1

Table Visuo-Spatial Functions

	A	B	C	D	E	F	G	H
1	P	Functions	Emis	MMSE	BELL	OCS	CLOCK (Mondini)	
2	1	M/L/C	Bilat	25,59			0	
3	2	M/L no*/C	Bilat	25,75			1	
4	3	M/L(*bilat or	Bilat	14,59			0	
5	4	M*no post/L	Bilat	28,1			1	
6	5	M/L	Left	29,49			1	
7	6	M/L/C*no pc	Right	29,99		1	0	
8	7	M* not done	Bilat	28,85			1	
9	8	M/L/C	Right	29,99	1	1	1	
10	9	M*no post /l	Left	27,5			1	
11	10	M/L/C*no pc	Left	27,75	1	1	1	
12	11	M (*R)/L	Left	25,97	1		1	
13	12	M/L/C (*only	Left	28,1	1		1	
14	13	M/L/C	Bilat	28,19			1	
15	14	M/L/C	Bilat	29,62	1	1	1	
16	15	M/L/N/C*No	Right	25,49	1		1	
17	16	M/L/C/N *N	Left	25,49	1	1	1	
18	17	M/L/C	Bilat	28,89	1		1	
19	18	M/L	Left	27,89			1	
20	19	M *No pre o	Left	28,49			1	
21	20	M/L	Bilat	28,31			1	
22	21	M/L/C	Bilat	28,31			1	
23	22	M/L/N/C*No	Bilat	26,59	1		1	
24	23	M/L/C	Bilat	28,75			1	
25	24	M/L/C*not p	Bilat	28,99			1	
26	25	M/L/C/N	Bilat	28,21	1		1	
27	26	M/L/C*only	Bilat	23,99	0	1	0	
28	27	M/L/C/N	Bilat	28,46	1	1	1	
29	28	M/L/C	Bilat	27,59	1		1	
30	29	M/L/C/N	Bilat	27,21	1		1	
31	30	M/L/N/C*No	Bilat	20,97	1	1	0	
32	31	M/L/C	Bilat	26,67			1	
33	32	M*no post o	Bilat	16,75	1	1	1	
34	33	M/L/C/N	Bilat	28,21	1	1	1	
35	34	M/L/C	Left	27,21			1	
36	35	M* not post	Bilat	29,49	1	1	1	
37	36	M/L/C	Left	28,75			1	
38	37	M/L/C	Left	28,21	1	1	1	
39	38	M/L/N/C*not	Bilat	17,89	1	1	0	
40	39	L/C/N	Bilat	28,07	1	1	1	
41	40	M/L/C	Bilat	28,21			1	
42	41	M/L/C	Bilat	28,21			1	
43	42	L	Bilat	24,99			1	
44	43	M/L/C/N	Bilat	28,75	1	1	1	
45	44	M/L/C/N(sol	Bilat	28,89		1	1	
46	45	M/L/C	Bilat	28,59			1	
47	46	M/L/C	Bilat	29,19			1	
48	47	M/L	Bilat	28,62	1		1	
49	48	M/L/C/N	Bilat	28,75			1	
50	49	M/L/C/N	Bilat	29,99	1	1	1	
51	50	M/L/C/N	Bilat	29,97	1	1	1	
52	51	M/L/C	Bilat	29,99	1	1	1	
53	52	M/L/C	Bilat	28,46	1	1	1	
54	53	M/L/C/N	Bilat	28,89			1	
55	54	M/L/C/N	Bilat	28,75	1	1	1	
56	55	M/L/C/N	Bilat	28,07	1	1	1	
57	57	M/L/C/N	Bilat	27,1			1	
58	58	M/L/C	Bilat	28,75			1	
59	59	M/L/C/N	Bilat	29,86	1	1	1	
60	60	M/L	Bilat	28,53	0		0	
61	61	M/L/C	Bilat	26,38			1	
62	62	M/L/C/N	Bilat	27,31	1	1	1	
63	64	M/L/C/N	Bilat	27,21	1	1	1	
64	65	M/L/C/N	Bilat	27,86	1	1	1	
65	66	M/L	Bilat	24,75	1	1	1	
66	67	M/L/C/N	Bilat	26,75	1	1	1	
67	68	M/L/C	Bilat	28,49	1	1	1	
68	69	M/L/C/N	Bilat	28,75	1	1	1	
69	70	M/L/C/N	Bilat	28,07	1	1	1	
70	71	M/L/C/N	Bilat	28,59				
